# H_2_FPEF score predicts atherosclerosis presence in patients with systemic connective tissue disease

**DOI:** 10.1002/clc.23621

**Published:** 2021-06-02

**Authors:** Vladimir Vasilev, Dejana Popovic, Gorica G. Ristic, Ross Arena, Goran Radunovic, Arsen Ristic

**Affiliations:** ^1^ School of Medicine, University of Belgrade Belgrade Serbia; ^2^ Division of Cardiology University Clinical Center of Serbia Belgrade Serbia; ^3^ Faculty of Pharmacy University of Belgrade Belgrade Serbia; ^4^ Department of Rheumatology and Clinical Immunology Military Medical Academy and Medical Faculty of the Belgrade Defence University Belgrade Serbia; ^5^ Department of Physical Therapy College of Applied Science, University of Illinois at Chicago Chicago Illinois USA; ^6^ Institute of Rheumatology Belgrade Serbia

**Keywords:** coronary artery disease, H_2_FPEF score, right ventricular–pulmonary vasculature uncoupling, systemic connective tissue disease

## Abstract

**Background:**

Cardiovascular diseases are common cause of morbidity and mortality in patients with systemic connective tissue diseases (SCTD) due to accelerated atherosclerosis which couldn't be explained by traditional risk factors (CVDRF).

**Hypothesis:**

We hypothesized that recently developed score predicting probability of heart failure with preserved ejection fraction (H_2_FPEF), as well as a measure of right ventricular‐pulmonary vasculature coupling [tricuspid annular plane systolic excursion (TAPSE)/pulmonary artery systolic pressure (PASP) ratio], are predictive of atherosclerosis in SCTD.

**Methods:**

203 patients (178 females) diagnosed with SCTD underwent standard and stress‐echocardiography (SE) with TAPSE/PASP and left ventricular (LV) diastolic filling pressure (E/e') measurements, carotid ultrasound and computed tomographic coronary angiography. Patients who were SE positive for ischemia underwent coronary angiography (34/203). The H_2_FPEF score was calculated according to age, body mass index, presence of atrial fibrillation, ≥2 antihypertensives, E/e' and PASP.

**Results:**

Mean LV ejection fraction was 66.3 ± 7.1%. Atherosclerosis was present in 150/203 patients according to: 1) intima‐media thickness>0.9 mm; and 2) Agatstone score > 300 or Syntax score ≥ 1. On binary logistic regression analysis, including CVDRF prevalence, echocardiographic parameters and H_2_FPEF score, only H_2_FPEF score remained significant for the prediction of atherosclerosis presence (χ^2^ = 19.3, HR 2.6, CI 1.5‐4.3, p < 0.001), and resting TAPSE/PASP for the prediction of a SE positive for ischemia (χ^2^ = 10.4, HR 0.01, CI = 0.01‐0.22, *p* = 0.004). On ROC analysis, the optimal threshold value for identifying patients with atherosclerosis was a H_2_FPEF score ≥2 (Sn 60.4%, Sp 69.4%, area 0.67, SE = 0.05, *p* < 0.001).

**Conclusions:**

H_2_FPEF score and resting TAPSE/PASP demonstrated clinical value for an atherosclerosis diagnosis in patients diagnosed with SCTD.

## 1 INTRODUCTION

Cardiovascular diseases (CVD) are the most common cause of morbidity and mortality in patients with systemic connective tissue diseases (SCTD) generally due to premature atherosclerosis.^1^ Current recommendations suggest assessment of general CVD risk factors (CVDRFs) in these patients, as part of risk prediction algorithms such as Systematic Coronary Risk Evaluation (SCORE)^2^ and Framingham^3^ in order to calculate a 10‐year risk of CVD events. These traditional risk factors include age, gender, blood pressure, smoking, diabetes mellitus, and hyperlipidemia and, according to calculated risk, preventive interventions are recommended.^2^, ^3^ However, in SCTD, atherosclerosis cannot be explained by traditional CVDRF alone.^1^, ^4^ The play a secondary role, while disease‐specific factors can directly influence the cardiovascular system. Chronic systemic inflammation and autoimmunity interfere in a number of metabolic processes, generating a proatherogenic condition.^1^, ^4^ In these patients, CVD is also influenced by anti‐inflammatory therapy.^1^, ^4^ Thus, the European League Against Rheumatism (EULAR) suggests screening, identification of CVDRF, and CVD risk management in all patients with SCTD.^1^ The EULAR task force has advocated the use of a 1.5 multiplication factor for these risk prediction models when certain rheumatoid arthritis (RA) disease characteristics were present.^1^ However, appropriate and validated SCTD‐specific CVD risk prediction models are still lacking.^1^ On the other hand, there is an urgent need to identify cardiovascular abnormalities early, before the development of irreversible damage.

It has been demonstrated that SCTDs are commonly associated with heart failure preserved ejection fraction (HFpEF) and pulmonary hypertension (PH),^5^, ^6^ increasing the risk of death by almost twofold.^7^ The synergy amongst HFpEF, systemic inflammatory disorders and atherosclerosis represents a vicious circle leading to very poor survival.^5^ As systemic inflammation precedes the onset of HFpEF and atherosclerosis by years,^8^ it would be beneficial to conduct early risk prediction in order to prevent future adverse events.

We hypothesized that the recently developed H_2_FPEF score, proposed to predict the probability of HFpEF through a composite score of six variables including age, body mass index (BMI), treatment with ≥2 antihypertensives, presence of atrial fibrillation, early diastolic filling pressure (E/e' ratio) and pulmonary artery systolic pressure (PASP) measured by echocardiography,^9^ is also predictive of atherosclerosis in patients diagnosed with SCTD. Moreover, we hypothesized that parameters of right ventricular‐pulmonary vasculature coupling (RV‐PV coupling) may also be predictive for atherosclerosis in this patient population. Thus, the purpose of the present investigation was to examine the predictive value of H_2_FPEF score and tricuspid annular plane systolic excursion (TAPSE)/PASP ratio, as a measure of RV‐PV coupling, for atherosclerosis presence in patients diagnosed with SCTD.

## 2 METHODS

### 2.1 Study cohort

From January 2018 to November 2019, 203 patients with SCTD (i.e., rheumatoid arthritis [RA], systemic lupus erythematosus [SLE], systemic sclerosis [SSc], and Sjogren's syndrome [SS]) diagnosed according to current criteria^10^, ^11^, ^12^, ^13^, ^14^, ^15^, ^16^, ^17^ were enrolled in this prospective observational study at the Clinical Center of Serbia. They were screened for study enrollment at the time of referral for a clinically indicated functional assessment. The study was approved by the local Ethical Institutional Review Board and informed consent was obtained from all subjects prior to enrollment. Clinical data from patients were collected during a preliminary visit, including age, gender, height, weight, BMI, and other CVDRFs (i.e., smoking status, hyperlipidemia, obesity, hypertension, heredity for CVD, diabetes mellitus). CVD risk was categorized as:(a) low; (b) intermediate; (c) high; and (d) very high according to SCORE value, determined using European Society of Cardiology (ESC) recommendations.^2^ Subjects afterwards underwent a two dimensional (2‐D) echocardiographic/Doppler evaluation, stress echocardiography (SE), computed tomographic coronary angiography (CTCA) and carotid ultrasound. Inclusion criteria were: (a) diagnosed RA, SLE, SSc, SS; (b) no previously documented CVD; (c) adequate echocardiographic windows; (d) left ventricular ejection fraction (LVEF) ≥ 50%; and (e) ability to exercise. Patients with normal LVEF and isolated tricuspid regurgitation due to a primary tricuspid valvular lesion were not included in the present investigation.^18^ Care was taken to identify the proper etiology of coexistent PH excluding idiopathic pulmonary arterial hypertension. Accordingly, we referred to Opotowsky et al.^19^ who proposed and validated 5‐point prediction score based on the measurements of LV diastolic filling pressure (E/e'), the antero‐posterior diameter of the left atrium and notching and/or shortened acceleration time of pulmonary flow. No subjects had significant right ventricular outflow tract obstruction.

### 2.2 Echocardiography

Echocardiographic imaging was performed using a Philips IE33 and a 5.2‐MHz transducer (Philips Medical Systems, Andover, MA) by two experienced cardiologists according to the current guidelines, at rest and during SE.^20^, ^21^ A 2‐D and Doppler examination was performed using a pre‐specified echocardiographic protocol by views specifically designed to optimize RV imaging.^22^ B–mode echocardiography was performed to assess LVEF and left atrial volume (LAV) at rest. Early (E) and late (A) LV diastolic filling velocity were assessed at rest and during SE. Tissue Doppler imaging (TDI) was recorded at rest and during SE at end‐expiration in the apical four chamber view at a sweep speed of 50 mm/s; the Doppler signal angle was less than 25%. Sample volume was positioned at 1 cm within the septal and lateral insertion sites of the mitral leaflets. Digitally stored loops of TDI were used for off‐line calculations of myocardial velocities. Average values of LV lateral and septal annular early diastolic filling velocities (e') were used to calculate E/e'. The apical four‐chamber view was used, and an M‐mode cursor was placed through the lateral tricuspid annulus in real time to obtain TAPSE at rest and during SE. The brightness was adjusted off‐line to maximize the contrast between the M‐mode signal arising from the tricuspid annulus and the background. TAPSE was measured as the total displacement of the tricuspid annulus (millimeters) from end‐diastole to end‐systole, with values representing TAPSE being averaged over three to five beats.^22^ PASP was estimated by Doppler echocardiography at rest and during SE from the systolic RV to right atrial pressure gradient using the modified Bernoulli equation. Right atrial pressure (assessed jugular venous pressure) was added to the calculated gradient to yield PASP. The TAPSE/PASP ratio, a measure of RV‐PV coupling,^23^ was calculated. Interobserver variability, assessed in a sample size of 20% of total population, was 3.5%, 3.4%, and 2.8% for M‐mode, 2‐D echocardiography and TDI, respectively.

### 2.3 Stress echocardiography

All subjects performed SE on a treadmill using the Bruce protocol according to established guidelines.^24^ Nitrates were stopped for 24 h, beta blockers for 3 days and calcium antagonists for 48 h before SE. Tea, coffee, cola‐drinks, chocolate, and smoking were not allowed for 24 h before the evaluation. Standard 12‐lead electrocardiograms were obtained after adequate skin preparation, at rest, each minute during exercise, and for at least 5 min during the recovery phase, according to established guidelines.^24^ Blood pressure was measured using a standard cuff sphygmomanometer. Test termination criteria consisted of: (a) symptoms (i.e., dyspnea and/or fatigue); (b) sustained ventricular tachycardia (VT) and non‐sustained VT that interfered with hemodynamic stability; (c) > 2 mm of horizontal or downsloping ST segment depression; (d) a drop of systolic blood pressure > 20 mm Hg during progressive exercise; (e) or reaching a submaximal heat rate (HR) calculated as 0.8·(220‐age). Wall motion was recorded at the beginning of the SE and at peak effort, and reported using a conventional 16‐segment model.^25^ An ischemic response (i.e., positive SE test) was defined as worsening LV wall motion during exercise testing in comparison to the resting condition.^25^

### 2.4 Coronary angiography

Coronary angiography was performed by the Judkins' technique.^26^ Stenosis was considered hemodynamically significant if there was a ≥ 50% reduction in luminal diameter. In order to assess the severity of CAD, the Syntax score was calculated.^27^ A Syntax score ≥ 1 was used to define the presence of atherosclerosis.

### 2.5 Computed tomographic coronary angiography

Patients received nitroglycerin 0.8 mg sublingually and metoprolol targeting a HR of ≤65 bpm before image acquisition. In order to calculate the time interval between intravenous contrast (Visipaque 320, GE Healthcare; Milwaukee, WI) infusion and image acquisition, a bolus tracking technique was used. A triphasic protocol was used for final image acquisition (100% contrast, 40/60% contrast/saline, and 40 cc saline). The infusion rate (5–6 cc/s) and contrast volume were individualized according to the patient's body habitus and scan time. GE high‐definition CT (VCL Lightspeed 64 MD, GE) was used to acquire retrospective ECG‐gated data sets with the width 64 mm × 0.625 mm slice collimation and a gantry rotation of 350 ms (mA = 300–800, kV = 120). Pitch (0.16–0.24) was individualized to the patient's HR. The CTCA data sets were reconstructed with an increment of 0.4 mm using the cardiac phase with the least cardiac motion. Images were interpreted by two radiologists blinded to all clinical data. The Coronary calcium accumulation ‐ Agatstone score (AS) was determined according to established guidelines.^28^ An AS >300 was used to define the presence of atherosclerosis.

### 2.6 Carotid ultrasound

Carotid ultrasound was performed by an experienced ultasonographer who was blinded to the clinical data, using ACUSON Antares System (Siemens AG, Munich, Germany) equipped with a 4–13 MHz linear array transducer, according to established guidelines.^29^, ^30^ Carotid intima media thickness (CIMT) was measured after the subject was placed in the supine position. The CIMT was defined as the distance between edges of the lumen‐intima and the media‐adventitia echoes, in a plaque‐free section. CIMT was measured bilaterally, at the levels of common carotid, carotid bifurcation and internal carotid artery. Mean values were calculated for all segments. According to the results of ARIC study, subclinical atherosclerosis was considered present if CIMT was higher than 0.9 mm.^31^, ^32^

### 2.7 Blood analysis

C‐reactive protein (CRP) was determined by particle enhanced immunoturbimetric assay (Tina‐quant CRP‐latex; Roche Diagnostic Corporation, Indianapolis, IN). The reference range for this assay is less than 5 mg/L.

### 2.8 Scoring

The H_2_FPEF score was calculated for each patient. The six variables that constitute the H_2_FPEF score are: (a) BMI >30 kg/m^2^ (2 points); (b) use of ≥2 antihypertensive medications (1 point); (c) history of atrial fibrillation (3 points); (d) PASP >35 mm Hg (1 point); (e) age > 60 years (1 point); and (f) E/e' > 9 (1 point).^9^

### 2.9 Statistical analysis

Continuous data are expressed as mean and standard deviation while categorical data are expressed as percentages. In order to apply parametric statistics, analysis of distribution was performed by the Kolmogorov–Smirnov test. The differences between the groups stratified according to presence of atherosclerosis were assessed by the Student's t test for independent samples. The Mann–Whitney test was used for nonparametric variables. Correlations between variables were performed by Pearson's correlation test and the Spearman's rank correlation test. Binary logistic regression analysis was performed to identify the best model to predict the probability of atherosclerosis. For the multivariate regression we used a forward conditional model with stepwise entry and removal criteria set at 0.05 and 0.10, respectively. Maximal iterations were set at 20. Hierarchical models were defined considering statistical significance and clinical relevance of independent variables, taking into consideration principal effects and second level interactions in each model. Measures of interest for predictive ability were compared using area under the receiver operating characteristic (ROC) curve. Two‐by‐two tables were built to estimate senstitivity (Sn), specificity (Sp), predictive values and 95% confidence intervals. Statistical tests were considered significant when a p‐value was <0.05 for all tests. The SPSS software package (SPSS version 27.0, SPSS Inc., Armonk, NY) was used for all analyses.

## 3 RESULTS

For 203 patients with SCTD enrolled, the mean age was 57.7 ± 11.2 years and 87.7% were female. The average time since diagnosis was 8.6 ± 7.9 years. Of the 203 subjects, 52 (25.6%) were diagnosed with RA, 51 (25.1%) with SLE, 50 (24.6%) with SSc and 50 (24.6%) with SS. Twenty eight of the subjects were only receiving symptomatic therapy (13.8%), 65 (32.0%) were receiving monotherapy (i.e. immunosuppressive drugs or biological therapy) and 110 (54.2%) were receiving combined specific therapy. The clinical data and the prevalence of CVDRF are given in Table S1.

All patients underwent SE testing; 34/203 (16.7%) tests were described as positive in terms of the presence of myocardial ischemia. All subjects with a positive SE test underwent catheterization and, of those catheterized,18/34 (52.9%) had significant lesions in the coronary arteries detected (Syntax score ≥ 1). Average Syntax score of all catheterized subjects was 8.3 ± 14.6. Of 18 patients with a Syntax score ≥ 1, five patients underwent a further revascularization procedure (two for percutaneous coronary intervention and three for coronary artery by pass graft).

CTCA and CIMT measurements were performed in all subjects; 40/203 (19.7%) had AS >300 and 139/203 (68.5%) had CIMT >0.9 mm. Average AS was 286.3 ± 696.3 and average CIMT was 1.2 ± 0.6 mm.

Subjects who demonstrated a AS >300, CIMT >0.9 mm or Syntax score ≥ 1 were arbitrarily considered to have documented atherosclerosis, as given in Table 1.

**TABLE 1:** Results of stress echocardiography, coronary angiography, computer tomographic coronary angiography, and carotid ultrasound

	Total	Positive	Negative
SE, n (%)	203/203 (100.0%)	34 (16.7%)	169 (83.3%)
Coronary angiography, n (%)	34/203 (16.7%)	18 (52.9%)	16 (47.1%)
CTCA, n (%)	203/203 (100%)	40 (19.7%)	163 (80.3%)
CIMT, n (%)	203/203 (100%)	139 (68.5%)	64 (31.5%)
Atherosclerosis, n (%)	203/203 (100%)	150 (73.9%)	53 (26.1%)

*Note*: Positive coronarography was considered Syntax score ≥ 1; positive CTCA AS >300, positive CIMT was considered CIMT >0.9 mm and positive atherosclerosis AS >300 or IMT > 0.9 mm or Syntax score ≥ 1.

Abbreviations: AS, Agatstone score; IMT, Intima‐media thickness; SE, stress echocardigraphy.

Echocardiographic data of patients with and without a positive SE test, as well as the presence or absence of atherosclerosis, are given in Table 2. RVDd, LAV, LAV index, resting E/e' and peak PASP were higher, while TAPSE/PASP at rest and peak were lower in subjects with positive SE and documented atherosclerosis. Patients with a positive SE test also had a lower LVEF and resting and peak TAPSE, whereas resting PASP was higher than in patients with a negative SE test. Patients with documented atherosclerosis had a lower resting and peak E/A and higher peak E/e' compared to patients with no documented atherosclerosis.

**TABLE 2:** Echocardiographic characteristics of study patients

Mean ± SD	Positive SE (n = 34)	Negative SE (n = 169)	*p* value	Documented atherosclerosis (n = 149)	No documented atherosclerosis (n = 54)	*p* value
EF, %	62.3 ± 9.4	67.2 ± 6.4	<.001***	66.1 ± 7.0	67.0 ± 7.7	.435
LVDd, mm	4.8 ± 0.7	4.8 ± 0.5	.972	4.8 ± 0.5	4.9 ± 0.5	.630
RVDd, mm	2.1 ± 0.4	2.0 ± 0.4	.033*	2.0 ± 0.4	1.9 ± 0.4	.033*
LAV, ml	35.8 ± 20.4	30.2 ± 11.0	.023*	32.8 ± 13.7	26.6 ± 10.4	.001**
LAV index, ml/m^2^	20.2 ± 11.0	16.6 ± 5.6	.050*	18.2 ± 7.2	14.5 ± 5.2	<.001***
VCI, mm	1.3 ± 0.4	1.3 ± 0.4	.906	1.3 ± 0.4	1.3 ± 0.3	.981
E/A rest	0.8 ± 0.3	0.9 ± 0.3	.290	0.9 ± 0.3	1.0 ± 0.4	.025*
E/A peak	1.0 ± 0.4	1.2 ± 1.6	.425	1.0 ± 0.4	1.7 ± 2.6	.003**
E/e' rest	10.4 ± 4.9	8.4 ± 2.6	.001**	9.1 ± 3.3	7.8 ± 2.6	.015**
E/e' peak	9.6 ± 4.4	8.8 ± 3.1	.229	9.2 ± 3.3	8.0 ± 3.2	.028*
PASP rest, mm Hg	39.5 ± 12.6	29.66 ± 6.4	<.001***	32.1 ± 9.2	29.7 ± 7.7	.167
PASP peak, mmHg	46.1 ± 17.7	34.7 ± 9.1	<.001***	38.2 ± 12.4	32.8 ± 9.9	.015*
TAPSE rest, mm	2.2 ± 0.4	2.3 ± 0.4	.025*	2.3 ± 0.4	2.3 ± 0.3	.603
TAPSE peak, mm	2.6 ± 0.4	2.8 ± 0.4	.005**	2.8 ± 0.4	2.8 ± 0.4	.867
TAPSE/PASP rest, mm/mm Hg	0.59 ± 0.20	0.83 ± 0.24	<.001***	0.76 ± 0.22	0.88 ± 0.31	.013*
TAPSE/PASP peak, mm/mm Hg	0.67 ± 0.38	0.87 ± 0.27	.002**	0.79 ± 0.30	0.94 ± 0.29	.011*

*Note*: Documented atherosclerosis considered Agatstone score > 300 or IMT > 0.9 mm or Syntax score ≥ 1.

Abbreviations: A, late diastolic filling velocity of the left ventricle; e', average of septal and lateral early left ventricular diastolic filling velocity measured by tissue Doppler; E, early diastolic filling velocity of the left ventricle; EF, ejection fraction; LAV, left atrial volume; LVDd, left ventricular end‐diastolic diameter; PASP, systolic pressure in pulmonary artery; RVDd, right ventricular end‐diastolic diameter; TAPSE, tricuspid annular plain systolic excursion; VCI, diameter of vena cava inferior.

*P < 0.05, **P < 0.01 and ***P < 0.001.

All patients had a LVEF ≥50%, except three patients that had a LVEF of 45%, which were excluded from further analysis. Mean H_2_FPEF score for patients with a LVEF ≥50% was 1.8 ± 1.4. H_2_FPEF score significantly correlated with the presence of atherosclerosis and SE positivity (*r* = 0.3, 0.2; *p <* .001, *p =* .005, respectively). H_2_FPEF score significantly correlated with TAPSE/PASP at rest and peak (*r* = −0.3, − 0.2; *p =* .001, .013, respectively).

On binary logistic regression analysis, CVDRF category, number of CVDRFs, VCI diameter, CRP and duration of illness were not significant predictors of atherosclerosis presence or a positive SE test. Significant predictors of both atherosclerosis presence and a positive SE test were RVDd, E/e' rest, LAV, LAV index, TAPSE/PASP at rest and peak, and H_2_FPEF score as given in Table 3 (A,B). LVEF was not a significant predictor of atherosclerosis presence, but significantly predicted a positive SE test. Peak E/e' significantly predicted atherosclerosis presence but did not have predictive value for a positive SE test. In a multivariate model, including all significant univariate predictors, only H_2_FPEF score remained in the regression for the prediction of atherosclerosis presence (*χ* ^2^ = 19.3, HR 2.6, CI 1.5–4.3, *p <* .001). When H_2_FPEF score was removed from the analysis, the only predictor that remained in the equation was peak TAPSE/PASP (*χ* ^2^ = 4.1, HR 0.2, CI 0.1–1.0, *p =* .049). In the multivariate model, the only predictor that remained in the regression for the prediction of SE positivity was resting TAPSE/PASP (*χ* ^2^ = 10.4, HR 0.01, CI 0.01–0.22, *p =* .004).

**TABLE 3:** Univariate and multivariate binary logistic regression analysis for key clinical and echocardiographic variables in the prediction of (A) atherosclerosis presence and (B) positivity of SE

(A)	*χ* ^2^	Hazard ratio	95% CI	*p* value
	Univariate analysis			
RVDd	4.2	2.5	1.0–5.9	.046*
LAV index	14.2	1.1	1.0–1.2	.001**
LAV	9.9	1.0	1.0–1.1	.035*
E/e'a rest	7.0	1.2	1.0–1.3	.015*
E/e'a peak	5.3	1.1	1.0–1.3	.031*
TAPSE/PASP rest	5.9	0.2	0.1–0.7	.017*
TAPSE/PASP peak	6.0	0.2	0.1–0.8	.016*
H_2_FPEF score	10.7	1.5	1.2–2.0	.002**
	Multivariate analysis			
H_2_FPEF score	19.3	2.6	1.5–4.3	<.001***

(B)	*χ* ^2^	Hazard ratio	95% CI	*p* value
	Univariate analysis			
EF	12.4	0.9	0.9–1.0	.001**
RVDd	4.4	2.7	1.1–6.9	.036*
LAV index	6.6	1.1	1.0–1.1	.010*
LAV	4.6	1.0	1.0–1.1	.030*
E/e'a rest	9.9	1.2	1.1–1.3	.003**
TAPSE/PASP rest	27.6	0.01	0.01–0.03	<.001***
TAPSE/PASP peak	12.6	0.04	0.01–0.29	.002**
H_2_FPEF score	6.1	1.4	1.1–1.9	.013*
	Multivariate analysis			
TAPSE/PASP rest	10.4	0.01	0.01–0.22	.004**

*Note*: Documented atherosclerosis considered Agatstone score > 300 or IMT > 0.9 mm or Syntax score ≥ 1.

Abbreviations: E, early diastolic filling velocity of the left ventricle; e'a, average of septal and lateral early left ventricular diastolic filling velocity measured by tissue Doppler; EF, ejection fraction; H_2_FPEF score, heart failure preserved ejection fraction score; LAV, left atrial volume; PASP, systolic pressure in pulmonary artery; RVDd, right ventricular end‐diastolic diameter; TAPSE, tricuspid annular plain systolic excursion.

*P < 0.05, **P < 0.01 and ***P < 0.001.

**FIGURE 1:**
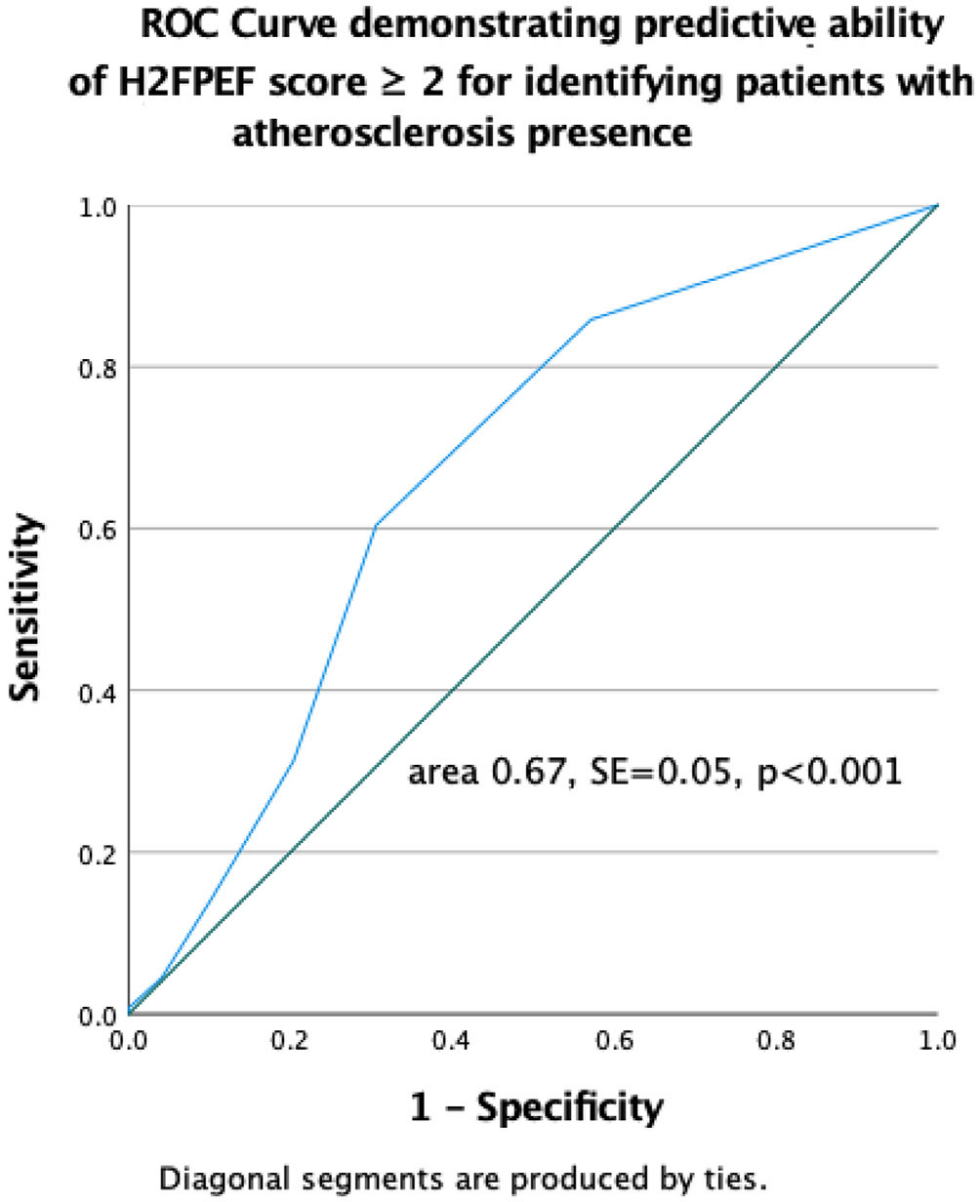
Receiver operating characteristic (ROC) curve demonstrating predictive ability of H_2_FPEF score ≥ 2 for identifying patients with atherosclerosis presence

In order to detect parameters to distinguish between those patients with and without atherosclerosis, ROC analysis was additionally used. The best predictive ability was shown for the H_2_FPEF score (area under ROC curve 0.67, SE = 0.05, *p <* .001). The optimal threshold value for identifying patients with atherosclerosis was a H_2_FPEF score ≥ 2, which produced a Sn and Sp of 60.4% and 69.4%, respectively, as shown in Figure 1. Predictive value was also shown for peak TAPSE/PASP (area under ROC curve 0.35, SE = 0.05, *p =* .004), as shown at Figure S1.

**FIGURE 2:**
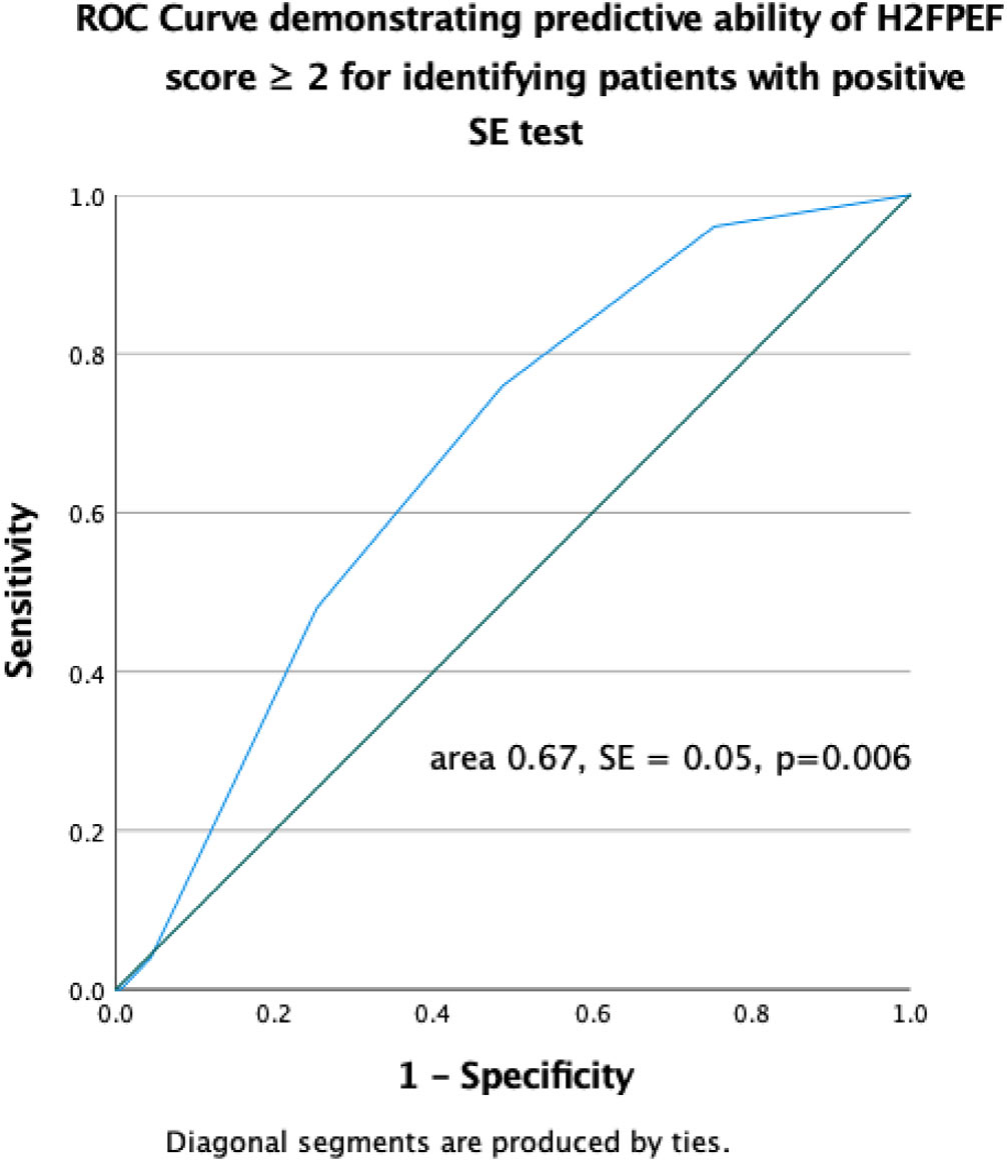
Receiver operating characteristic (ROC) curve demonstrating predictive ability of H_2_FPEF score ≥ 2 for identifying patients with positive SE test

On ROC analysis, H_2_FPEF score was also shown to have a predictive value for identifying patients with positive and negative SE test (area under ROC curve 0.67, SE = 0.05, *p =* .006). The optimal threshold value for identifying patients with a positive SE test was a H_2_FPEF score ≥ 2, which produced a Sn and Sp of 76.0% and 48.7%, respectively, as shown in Figure 2. A predictive value in distinguishing between a positive and negative SE test was also shown for resting TAPSE/PASP (area under ROC curve 0.21, SE = 0.05, *p <* .001), as shown at Figure S2.

## 4 DISCUSSION

The present findings demonstrate that more than two thirds of patients with SCTD had occult atherosclerosis. Patients with atherosclerosis demonstrate a worsened echocardiographic phenotype as indicated by a larger RV and LA, higher LV diastolic filling pressures at rest and during exercise, as well as a lower resting and peak TAPSE/PASP, suggesting worse RV‐PV coupling. The recently developed H_2_FPEF score, a powerful tool in predicting HFpEF presence,^9^ outperformed all other measures assessed in the present study, including the ESC CVD risk categorization, in the prediction of atherosclerosis presence. A high predictive value for atherosclerosis presence and development of myocardial ischemia in patients diagnosed with SCTD was also demonstrated for rest and peak TAPSE/PASP, and they both significantly correlated with H_2_FPEF score.

Accumulating evidence points to the existence of increased CVD risk in patients diagnosed with SCTD in comparison to the general population, particularly in patients with RA, whose risk is comparable with diabetes mellitus.^32^, ^33^, ^34^ Studies show that the risk of myocardial infarction is approximately 70% higher in these patients than in general population.^35^ Early recognition of CVD risk in SCTD patients is of a great clinical importance, as that would prevent future adverse events and poor survival. There is a need to find an easy method, feasible not only for experts in cardiology and internal medicine, but every other physician involved in care of patients diagnosed with SCTD. In addition to standard CVDRFs which are emphasized in SCTD, such as physical inactivity due to functional disability, common concomitant hypothyroidism, diabetes mellitus, hyperlipidemia, chronic kidney disease and hypertension, there are novel risk factors playing a role in this condition, such as inflammation and applied therapy.^1^ Drugs with powerful suppression of inflammation may slow down progression of atherosclerosis.^1^ There is accumulating evidence that decreasing the inflammatory burden, by biologic therapy, RA patients translates into a lower CVD risk.^1^ Besides RA, no other SCTD is currently incorporated into any widely accepted CVD risk calculators.^2^ This is not appropriate approach, as accelerated atherosclerosis and heart failure coexist in all distinct phenotypes of SCTD.^5^, ^32^, ^34^, ^36^ The mechanisms that contribute to accelerated atherosclerosis are still incompletely understood. Chronic systemic inflammation and autoimmunity interfere in a number of metabolic processes, influencing liver function, skeletal muscles, and fat tissue, generated a proatherogenic condition.^1^, ^4^ The proinflammatory cytokines, such as tumor necrosis factor (TNF)‐α and interleukin (IL)‐6, lead to endothelial dysfunction and activation, primarily in patients with RA.^1^ Enhanced expression of adhesion molecules (such as vascular cell adhesion protein 1, or VCAM‐1) on the endothelial and smooth muscle cell surfaces is associated with cardiovascular disorders in RA and SLE.^4^ In SLE patients CVD are generally independent of traditional risk factors and SLE itself has been shown to be a substantial risk factor for the development of atherosclerosis.^4^, ^35^ In addition to the role of inflammation, patients with SLE have antinuclear antibodies which might have a pathophysiological role.^4^ The underlying mechanisms for atherosclerosis in patients with SSc involve endothelial injury and reduced oxygen transport to tissues. SSc (both limited and diffuse disease) is also associated with increased stiffness of the vasculature.^7^ Although RA exhibited the highest CVD risk, the one is only slightly lower in SSc, SLE, and SS.^1^, ^33^, ^36^ This observation was encouraging to examine all these SCTD in the current study. Although highly recommended to determine,^1^, ^2^ SCORE CVD risk evaluation in patients with SCTD underestimates real CVD risk.^1^ Even with a deeper diagnostic approach suggested by guidelines, including screening for asymptomatic atherosclerotic plaques through carotid ultrasound, CVD risk estimation is still based on expert opinion.^1^, ^2^ Previous evidence indicates SCTD leads to myocardial inflammation, abnormalities of coronary microcirculation, diastolic dysfunction, LA enlargement, and finally HFpEF.^7^ Interestingly, women have greater risk and suffer greater cardiac consequences from these systemic inflammatory and metabolic disorders.^5^ Coronary artery calcifications, a marker of coronary atherosclerosis, are shown to stratify risk of HFpEF beyond traditional risk factors in women.^29^ Accordingly, a new marker was recently identified, an inflammatory‐metabolic phenotype of HFpEF, characterized by biomarkers of inflammation, microvascular endothelial dysfunction, normal‐to‐mildly increased left ventricular volumes and systolic blood pressures, and altered activity of adipocyte‐associated inflammatory mediators.^5^ This HFpEF phenotype may express independent of large vessel coronary artery disease.^5^ The proposed underlying pathophysiology for this condition is an inflammatory response to an ectopic accumulation of dysfunctional lipids, in the epicardium or small coronary vessels, leading to fibrosis of the adjacent myocardium, ventricular and atrial, with consequent impairment of diastolic function and atrial fibrillation.^5^, ^30^, ^31^ This sequence could explain the link between accelerated atherosclerosis in SCTD and HFpEF development.^31^ In support, our study demonstrated larger LA, and higher LV diastolic filling pressures at rest and during effort in SCTD subjects with positive SE and documented atherosclerosis, but also the impact on RV function and worsening RV‐PV coupling, which is an increasingly important feature in HFpEF.^32^ Previous studies have demonstrated that right heart function is a crucial determinant of outcome in HF patients regardless of LV function or predominance of systolic or diastolic HF.^33^ In atherosclerotic patients, abnormalities in PASP and PVR are observed with a direct link to incident HFpEF and it is proposed that impairments in PV function may precede clinical HFpEF.^34^ Accordingly, the link between TAPSE/PASP as a measure of RV‐PV coupling and RV function was shown to provide a comprehensive tool for risk stratification in HFpEF.^32^ In our study, the TAPSE/PASP response demonstrated a high predictive value for atherosclerosis presence and positive SE test, outperforming other LA and LV parameters, suggesting the importance of RV and pulmonary circulatory dysfunction in atherosclerotic patients with SCTD. The significant correlation of TAPSE/PASP with the H_2_FPEF supports the link of RV‐PV uncoupling and HFpEF, which is already very well known.^35^

Nonetheless, a H_2_FPEF score was superior to TAPSE/PASP both at rest and peak exercise in the prediction of atherosclerosis presence, supporting its' comprehensive nature in assessing clinical status. Considering the potential presence of both HFpEF and atherosclerosis in SCTD patients, the strong predictive value of the H_2_FPEF score is not surprising, as all components of the score are in a way a reflection of underlaying pathophysiology. Some previous studies have demonstrated that a high H_2_FPEF score may be associated with a high SYNTAX score and may be used to estimate the extent and complexity of coronary artery disease.^36^ Simplicity of the determination and calculation of the H_2_FPEF score is of a particular value from a clinical perspective, which may help planning further diagnostic procedures in the evaluation of SCTD patients with suspected atherosclerosis.

## 5 LIMITATIONS

A limitation of this study is the lack of invasive hemodynamic evaluation for HFpEF presence. Sub analyses on age and gender were not performed due to the limited numbers in specific subsets of patients, which should be addressed in future studies. Moreover, to define the presence of atherosclerosis, the cut off values were arbitrarily chosen. While CIMT >0.9 mm is commonly used and easily understood by most clinicians^37^, the impact of age and sex cannot be neglected.^38^ Furthermore, CIMT measurements were usually performed at the common carotid artery, however previous studies differed according to measurement side (left side versus right side), measurement wall (far wall versus near wall) and method of combination of single measurements (mean versus mean‐maximum).^39^, ^40^ The use of different ultrasound machines with varying transducer frequencies may also be impactful.^39^, ^40^ Syntax score ≥ 1 is another cut off determinant that could be understood as too low, however, the intention was to identify any presence of atherosclerosis, regardless of its significance. The cut off for AS >300 was chosen as it was shown previously to be associated to markedly increased CVD risk, however that risk was observed in patients with AS >100 as well.^41^ Another limitation of the current study is low H_2_FPEF score observed in study population. Future studies with more severe patients and higher score are warranted.

## 6 CONCLUSIONS

The H_2_PHEF score has strong predictive value for atherosclerosis presence in patients diagnosed with SCTD. The same was shown for TAPSE/PASP measured at rest and during peak exercise, novel unfavorable markers of RV‐PV uncoupling and RV dysfunction, which correlate with the H_2_PHEF score. Present findings indicate the need to systematically calculate H_2_PHEF score in patients with SCTD in order to help reveal occult atherosclerosis.

## CONFLICT OF INTEREST

The authors declare no potential conflict of interest.

## Supporting information


**Figure S1**: ROC curve demonstrating predictive ability of TAPSE/PASP peak for identifying patients with atherosclerosis presence.

**Figure S2**: ROC curve demonstrating predictive ability of TAPSE/PASP rest for identifying patients with positive SE test.

**Table S1**: Clinical characteristics of study population.

## Data Availability

Research data are not shared.
