# Supplementary figures and images for: H2FPEF score predicts atherosclerosis presence in patients with systemic connective tissue disease
Source: Clin Cardiol. 2021 Jun 2;44(7):946–54. doi: 10.1002/clc.23621 (PMC8259163; doi:10.1002/clc.23621)

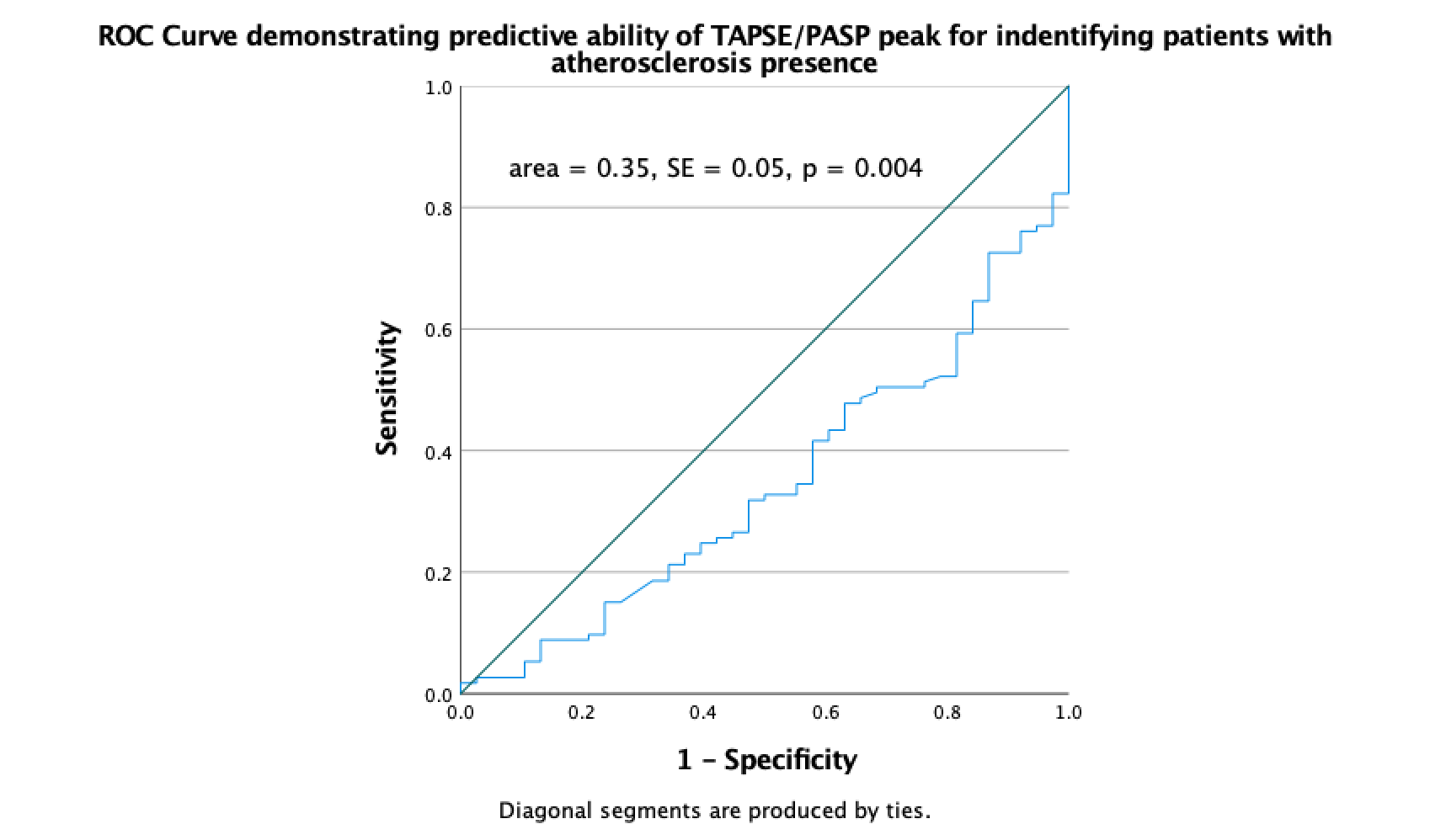

Supplement: Supplementary file 1 — Figure S1: ROC curve demonstrating predictive ability of TAPSE/PASP peak for identifying patients with atherosclerosis presence. [file CLC-44-946-s001.tif]

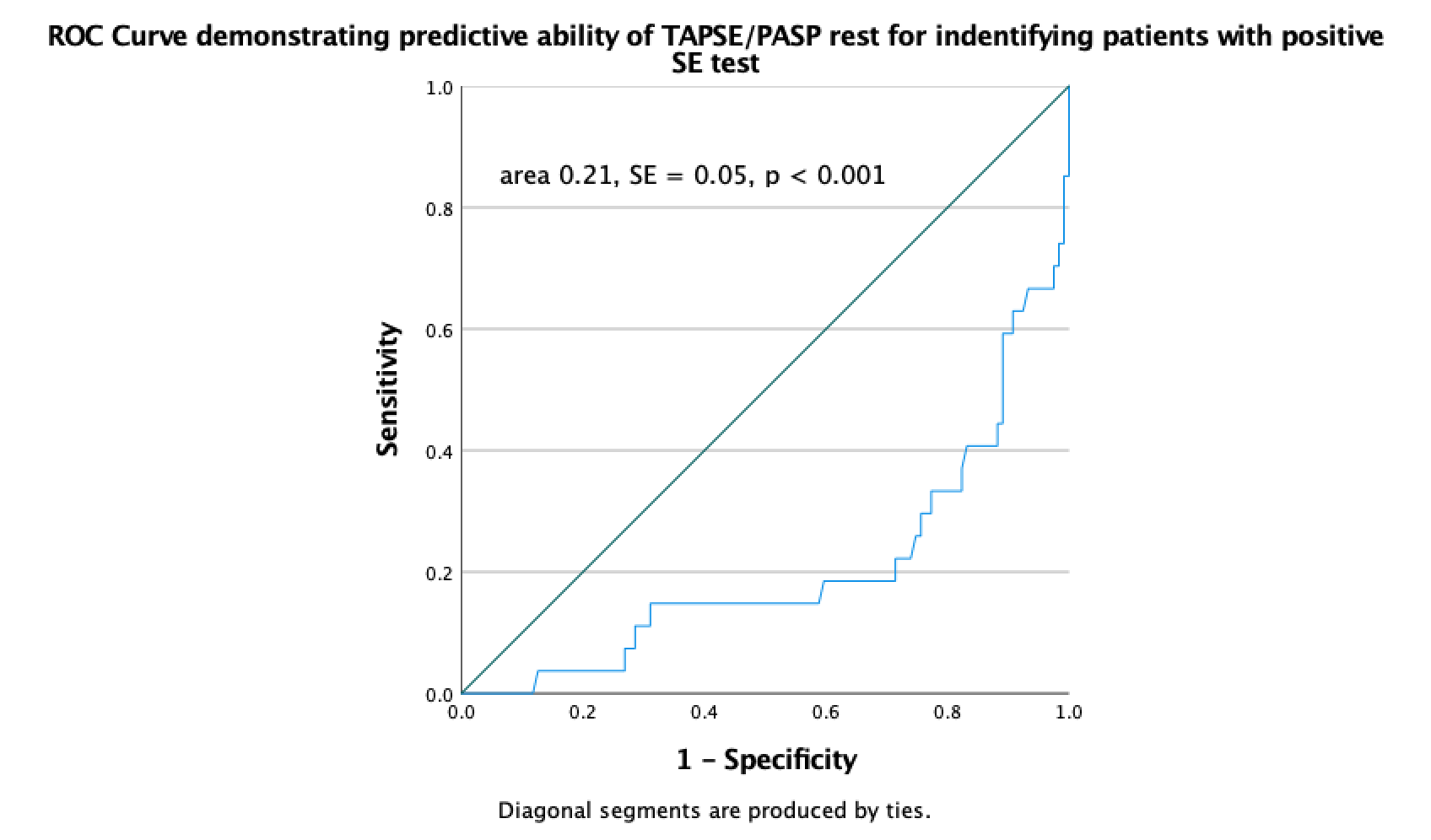

Supplement: Supplementary file 2 — Figure S2: ROC curve demonstrating predictive ability of TAPSE/PASP rest for identifying patients with positive SE test. [file CLC-44-946-s003.tif]
