# Supplementary material for: H2FPEF score predicts atherosclerosis presence in patients with systemic connective tissue disease
Source: Clin Cardiol. 2021 Jun 2;44(7):946–54. doi: 10.1002/clc.23621 (PMC8259163; doi:10.1002/clc.23621)
Supplement: Supplementary file 3 — Table S1: Clinical characteristics of study population. [file CLC-44-946-s002.docx]

**Table 1S.** **Clinical characteristics of study population**

|  | **Mean** | **±** | **SD** |
| --- | --- | --- | --- |
| **Age (years)** | 57.7 | **±** | 11.2 |
| **BMI (kg/m^2^)** | 25.9 | **±** | 4.6 |
| **SBP (mmHg)** | 132.9 | **±** | 18.5 |
| **DBP (mmHg)** | 84.3 | **±** | 12.4 |
| **HR (beats/min)** | 80.4 | **±** | 14.5 |
| **CRP (mg/L)** | 1.7 | **±** | 0.8 |
| **Group** | **n (%)** | | |
| **RA** | 52 (25.6%) | | |
| **SLE** | 51 (25.1%) | | |
| **SSc** | 50 (24.6%) | | |
| **SS** | 50 (24.6%) | | |
| **Female** | 178 (87.7%) | | |
| **Atrial fibrillation** | 2 (1.0%) | | |
| **Smoking** | 81 (39.9%) | | |
| **Hyperlipidemia** | 45 (22.2%) | | |
| **Obesity** | 67 (33.0%) | | |
| **Hypertension** | 109 (53.7%) | | |
| **Heredity for CVD** | 95 (46.8%) | | |
| **Diabetes** | 19 (9.4%) | | |
| **Low ESC CVDRS** | 102 (50.2%) | | |
| **Moderate ESC CVDRS** | 49 (24.1%) | | |
| **High ESC CVDRS** | 47 (23.2%) | | |
| **Very High ESC CVDRS** | 5 (2.5%) | | |
| **Therapy** |  | | |
| Symptomatic therapy | 28 (13.8%) | | |
| Monotherapy | 65 (32.0%) | | |
| Combined therapy | 110 (54.2%) | | |
| **Medications** |  | | |
| Corticosteroids | 94 (46.3%) | | |
| NSAID | 12 (5.9%) | | |
| Antimalarial drugs | 18 (8.9%) | | |
| Immunosuppressive therapy | 49 (24.1%) | | |
| Biological therapy | 15 (7.4%) | | |
| Beta blockers | 82 (40.4%) | | |
| ACE I/ARB | 77 (37.9%) | | |
| CCB | 55 (27.1%) | | |
| Diuretic | 47 (23.2%) | | |
| Statins | 36 (17.7%) | | |

ACE I = Angiotensin converting enzyme inhibitor, ARB = Angiotensin receptor blocker, BMI = Body mass index, CCB = Calcium channel blocker, CRP = C-reactive protein, CVDRS = Cardiovascular Disease Risk Score, DBP = Diastolic blood pressure, ESC = European Society of Cardiology, HR = Heart rate, NSAID = Non-steroid anti-inflammatory drugs, RA = Rheumatoid arthritis, SBP = Systolic blood pressure, SLE = Systemic lupus erythematosus, SSc = Systemic sclerosis, SS = Sjogren syndrome
